# Measuring coverage of maternal and child health services using routine health facility data: a Sierra Leone case study

**DOI:** 10.1186/s12913-021-06529-7

**Published:** 2021-09-13

**Authors:** Abdoulaye Maïga, Agbessi Amouzou, Moussa Bagayoko, Cheikh M. Faye, Safia S. Jiwani, Dauda Kamara, Ibrahim B. Koroma, Osman Sankoh

**Affiliations:** 1 Bloomberg School of Public Health, Department of International Health, Johns Hopkins University, 615 N Wolfe St. 21205, Baltimore, USA; 2 African Population and Health Research Center, Nairobi, Kenya; 3 Ministry of Health and Sanitation, Freetown, Sierra Leone; 4 Statistics Sierra Leone, Freetown, Sierra Leone; 5 Njala University, University Secretariat, Njala, Moyamba, Sierra Leone; 6 School of Public Health, Faculty of Health Sciences, University of the Witwatersrand, Johannesburg, South Africa; 7 Heidelberg Institute for Global Health, University of Heidelberg Medical School, Heidelberg, Germany

**Keywords:** RHIS data, Measuring coverage, Data quality, Maternal and child health, Sierra Leone

## Abstract

**Background:**

There are limited existing approaches to generate estimates from Routine Health Information Systems (RHIS) data, despite the growing interest to these data. We calculated and assessed the consistency of maternal and child health service coverage estimates from RHIS data, using census-based and health service-based denominators in Sierra Leone.

**Methods:**

We used Sierra Leone 2016 RHIS data to calculate coverage of first antenatal care contact (ANC1), institutional delivery and diphtheria-pertussis-tetanus 3 (DPT3) immunization service provision. For each indicator, national and district level coverages were calculated using denominators derived from two census-based and three health service-based methods. We compared the coverage estimates from RHIS data to estimates from MICS 2017. We considered the agreement adequate when estimates from RHIS fell within the 95% confidence interval of the survey estimate.

**Results:**

We found an overall poor consistency of the coverage estimates calculated from the census-based methods. ANC1 and institutional delivery coverage estimates from these methods were greater than 100% in about half of the fourteen districts, and only 3 of the 14 districts had estimates consistent with the survey data. Health service-based methods generated better estimates. For institutional delivery coverage, five districts met the agreement criteria using BCG service-based method. We found better agreement for DPT3 coverage estimates using DPT1 service-based method as national coverage was close to survey data, and estimates were consistent for 8 out of 14 districts. DPT3 estimates were consistent in almost half of the districts (6/14) using ANC1 service-based method.

**Conclusion:**

The study highlighted the challenge in determining an appropriate denominator for RHIS-based coverage estimates. Systematic and transparent data quality check and correction, as well as rigorous approaches to determining denominators are key considerations to generate accurate coverage statistics using RHIS data.

**Supplementary Information:**

The online version contains supplementary material available at 10.1186/s12913-021-06529-7.

## Background

Household surveys and routine health information systems (RHIS) are critical sources of coverage data for health programs planning, monitoring and performance assessments in low- and middle-income countries [1–4]. Yet, national household surveys such as Demographic and Health Survey (DHS) or Multiple Indicators Cluster Survey (MICS) which represent the main source of coverage data are costly, resources-intensive and not conducted regularly in all countries. For instance, the most recent DHS or MICS was carried out more than a decade ago in Burkina Faso, Central African Republic, Cabo Verde. In addition, these national surveys do not generate estimates at subnational levels below the first administrative level. Routine health facility data, on the other hand, are collected monthly and can be disaggregated to smaller administrative levels like districts or health facility catchment area. These sources have however been underutilized due to concerns about data quality, completeness, representativeness, and adequate methods to calculate estimates [5, 6].

Good quality RHIS is an essential component of a strong health system providing continuous and timely data for decision-making across all the other health system functions (service delivery, health workforce, access to essential medicines, financing, leadership, and governance) locally and nationwide [7]. Given their potentials and the recent effects of the Covid-19 pandemic on household data collection, there is an increased global commitment and country interest in improving and using these data to produce health statistics and coverage estimates [8–11]. The introduction of District Health Information Software 2 (DHIS2) [12, 13] has contributed to improving data collection and quality checks. However, large efforts remain to substantially improve data quality in most countries [6, 10, 14].

A critical challenge in using RHIS data for coverage statistics is the correct measurement of the denominators. While RHIS provides the numerator – those who receive a service – it is essential to accurately estimate the population in need of the service in order to arrive at a reliable coverage measure [2]. Methods for estimating the denominators are traditionally based on population projections. The use of health facility data itself to indirectly derive denominators for selected coverage indicators is only recently and less common, since data quality remains questionable and the calculation requires a rather complex procedure [2, 6, 10].

The objective of this study was to generate coverage estimates from RHIS data and assess their accuracy for selected maternal and child health indicators in Sierra Leone. We focused on the coverage of at least one antenatal care contact (ANC1), institutional delivery, and the third dose of diphtheria-pertussis-tetanus (DPT3) immunization. Data availability and quality as well as consideration of key coverage indicators in maternal and child frameworks explain the choice of these indicators [15–18]. We carried out the assessment at national and district levels and compared RHIS-based coverage estimates to the 2017 MICS used as benchmark.

## Methods

### Study context and data

This study was based on results from a one-week capacity strengthening workshop on health facility data analysis conducted in Dakar (Senegal) in 2019 by the Countdown to 2030 collaboration [19]. It involved RHIS officers and analysts from national research and statistical institutes from twenty West and Central African countries (Table A1 in annex) [19]. Using a pre-designed template, country participants compiled facility data from their countries, which included indicators of service provision on antenatal care, deliveries, child immunization covering the period 2014–2018. Table A2 in annex includes the list of indicators in the RHIS data analyzed based on data availability and quality. We focused this case-study on RHIS data from Sierra Leone which included subnational data for which household survey coverage estimates were also available from the 2017 MICS [20] for comparison for the same reference period. Sierra Leone had the most recent census data (2015) and the same administrative units (districts) were used for both the RHIS and the MICS. It should be noted that Sierra Leone created in July 2017 two additional districts (Falaba and Karena) after the MICS exercise. Moreover, RHIS data were collected for the two new districts only from March 2020. The study was therefore based on the original 14 old districts of the country due to the lack of RHIS disaggregated data for the 16 districts and the methodological orientation of the analysis.

Sierra Leone is a West African country with a total population of 7,092,113 inhabitants in 2015 and an average annual growth rate of 3.2% over the past 10 years. The annual population growth at subnational level ranged from 1.8 to 8.5% [21]. The DHIS2 platform was introduced since 2009 and WHO data quality module was incorporated. However, the system was paper-based at facility level and data were entered into the web-database at district level. The demographic and population projection data used by the RHIS were extracted from publicly available official publications of Statistics Sierra Leone [22] – the national institute of statistics.

For country selection, we carried out data quality checks for all the countries that participated in the workshop (see Tables A1, A3, A4, and Figure A1 in annex). Table 1 describes data quality checks for Sierra Leone at national and districts levels using WHO-recommended data quality metrics [23]. These include completeness of reporting, identification of major outliers, consistency over time, and internal consistency between ANC1 and DPT1 and between DPT1 and DPT3 services data. We calculated a quality score for each metric as well as an average total score (Table 2).

**Table 1:** Data quality checks for Sierra Leone RHIS data

The data quality assessment was based on quality checks manuals, guidelines and studies published elsewhere [2, 6, 10, 23, 24]. We created and used an Excel Spreadsheet Dashboard to assess data quality. We made this tool which includes formulas and instructions for quality checks publicly available [25]. We computed the average score of completeness of reporting [23] including percentage of reporting for ANC, delivery and immunization services. The completeness of reporting was 92% and above the minimum threshold of 80% suggested by WHO [23], but there were differences across districts. Incomplete reportings were noticeable in Western Area Rural and Western Area Urban districts (completeness = 81%) while a few districts reportings had completeness over 97% (Kambia, Moyamba, Kenema, Koinadugu) (Table 2). We assessed major outliers of monthly aggregated data in 2016 for each district and nationwide. For each monthly data, we calculated a modified Z-score which is a standardized score of observations measuring the deviation from the median. It was computed by dividing the difference from the median by the median absolute deviation. The modified z-score is a robust statistic for small samples as compared to the traditional Z-score. Monthly data with a score higher than 3.5 standard deviation (SD) from the annual median were identified as outliers [23, 24]. The analysis identified two districts (Kono and Moyamba) with over 12% of reported data as outliers. Outliers do not always reflect data quality issues, but may be due to factors such as stock-outs, population migration, seasonality of service/care seeking, or other contextual factors during the months identified as potential outliers [10, 23]. We checked the internal consistency of data over time, by assessing whether the year 2016 can be considered as an outlier compared to years 2015, 2017 and 2018. We calculated a modified-Z score for ANC1 and DPT1 services by district and nationally, and the average score expressed in percentage [23, 24]. A year-to-year variation can be expected as a result of population growth and changes in service utilization, but this should be limited and the reported numbers consistent over time. Half of the districts had a score 70% or higher. Highest inconsistencies in time-series data were noticeable in Kailahun, Kenema, Koinadugu, and Western Area Urban districts. The internal consistency between ANC1 and DPT1 service and between DPT1 and DPT3 service was the fourth quality metric. For ANC1 and DPT1, we calculated a quality score as the absolute difference between the expected and the reported ratios of the two indicators. The expected ratio was obtained from the most recent household survey. We used a similar process to assess the consistency of DPT1 and DPT3 services, and then expressed the differences into scores. The high quality score nationwide (mean = 82%; median = 88%) however concealed consistency issues in districts like Bo (25%) and Western Area Urban (50%).

**Table 2:**
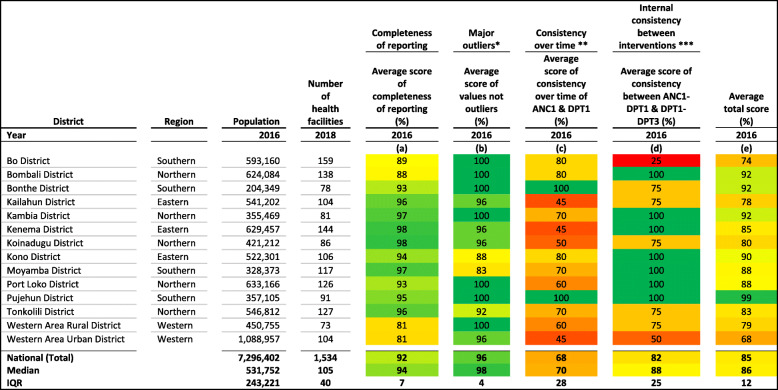
Data quality checks metrics – completeness of reporting, outliers, consistency over time and consistency between interventions

(a) Average percentage of completeness stands for average percentage of ANC1, Delivery, and BCG/DPT/Penta completeness of reporting; (b) Average percentage of outliers for ANC1 and DPT3; (c) Average percentage for ANC1 and DPT3; (d) Average percentage for consistency pair ANC1-DPT1 and pair DPT1-DPT3; (e) Average percentage of (a), (b), (c) and (d)

* Outliers defined as modified z-score greater than 3.5 for monthly reported data

** Assigned quality score to modified z-score based on cut-off values (< 0.25, 100%; > = 0.25 & < 0.5, 80%; > = 0.5 & < 0.75, 60%; > = 0.75 & < 1, 50%; > = 1 & < 1.25, 40%; > = 1.25 & < 1.5, 30%; > = 1.5 & < 1.75, 20%; > = 1.75 & < 2, 10%; > = 2, 0%). The quality score was divided up into deciles for the bottom 60% and into quartiles for the top 40%

*** Percentage difference between routinely reported ratio and survey ratio: values were classified as (<=5, 100%; > 5 & < 15, 75%; > = 15 & < 20, 50%; > = 20 & < 25, 25%; > = 25, 0%). The bottom quartile of the quality score was arbitrary divided up into five sub-groups

*ANC* antenatal care; *BCG* Bacille de Calmette and Guerin; *DPT* diphtheria-pertussis-tetanus

Color scale indicates good data quality for green color while red color corresponds to poor data quality

### Adjustment for incomplete reporting

We calculated all the statistics based on adjusted numbers (N_adj_) accounting for incomplete reporting using Maina and colleagues’ method [2]. This was done using the reported number (N_rep_), the completeness of facility reports (c) and an adjustment factor (k) that reflects the assumed level of services from non-reporting facilities.

$$ {N}_{adj}={N}_{rep}+{N}_{rep}\ast \left(\frac{1}{c}-1\right)\ast k $$

The reported number and completeness of reporting were available from RHIS data whereas the adjustment k-factor had to be determined. A k-factor of 0 means that no services were provided by non-reporting facilities while a value of one indicates the same level of services in the non-reporting facilities. In Sierra Leone where the completeness of reporting was 94, 89 and 94% for ANC, vaccination and delivery service respectively, RHIS officers and country analysts recommended a k-value of 0.25 for all services. The assumption being that some services were provided by non-reporting facilities, but the level of service was lower and expected to be equal to 25% of service provision in reporting facilities.

### Coverage measurement

The calculation of a service coverage requires the number of individuals who actually received the service (numerator) and the total population who need the service (denominator). The numerators were the number of ANC1, deliveries and DPT3 reported by facilities which were adjusted for incomplete reporting according to the adjustment approach previously described. Each of the denominators was calculated according to two census-based methods as well as three health service-based methods.

The first census-based approach uses the projected total population from the most recent census and the crude birth rate (CBR) to derive the total live births. The CBR was obtained from the latest population-based survey. The expected total number of births, deliveries and pregnancies were estimated by applying the stillbirth rate, the proportion of multiple births (twins, triplets) and the proportion of pregnancies ending in early fetal death. We used the expected number of live births and the neonatal mortality rate to calculate the expected number of infants. The second census-based method directly uses the projected number of live births from the recent population census. (see Figure A2 in annex).

We used the reported number of BCG, ANC1 and DPT1 to derive the three health service-based denominators (see Figure A3 in annex). The reported numbers were adjusted both for incomplete reporting and for non-use of service to get the expected number of pregnancies. The percentage of non-use of service was estimated from the most recent household survey. Similar adjustments were made to estimate the expected number of total pregnancies, deliveries, births, and infants as in the case of the census-based approaches.

### Assessment of coverage estimates

**Fig. 1:**
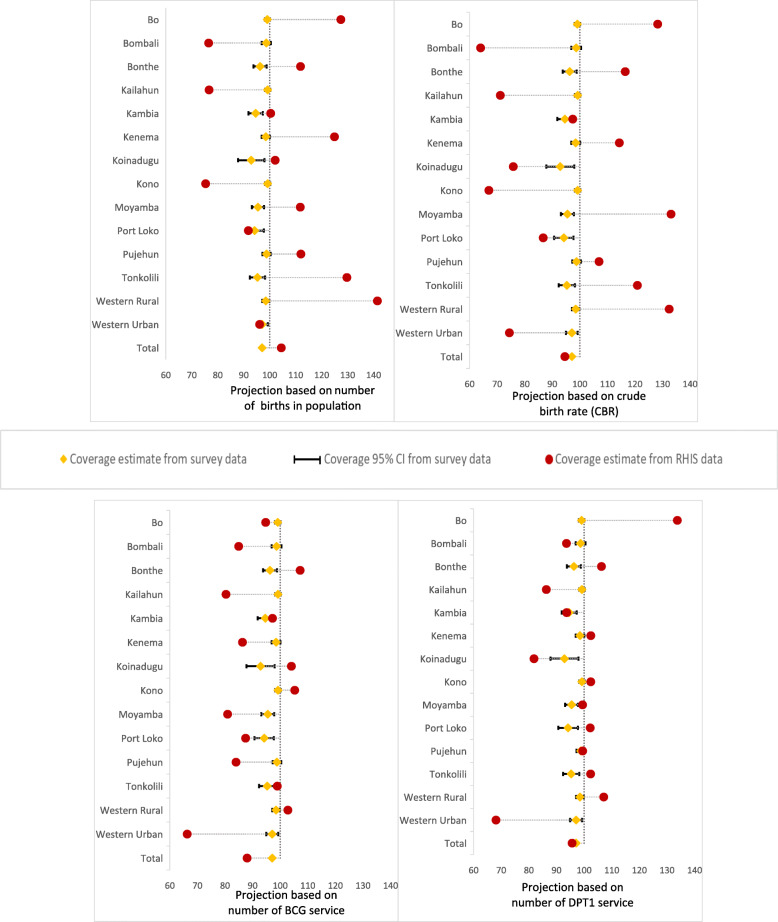
Level of agreement of ANC1 coverage estimates (%) between RHIS data and 2017 MICS, by district, using four different methods for calculating denominators

**Fig. 2:**
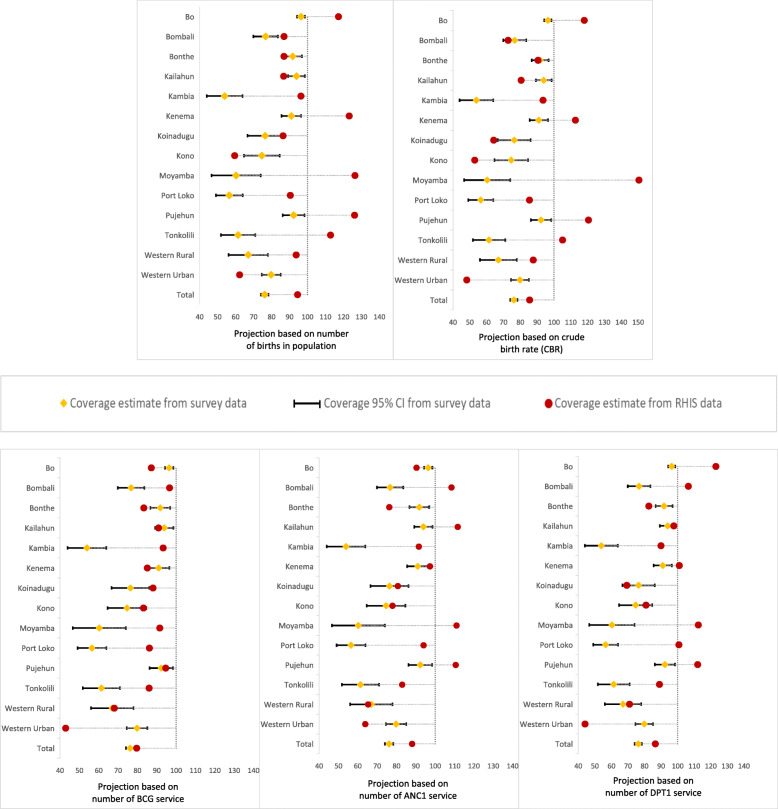
Level of agreement of institutional delivery coverage estimates (%) between RHIS data and 2017 MICS, by district, using five different methods for calculating denominators

**Fig. 3:**
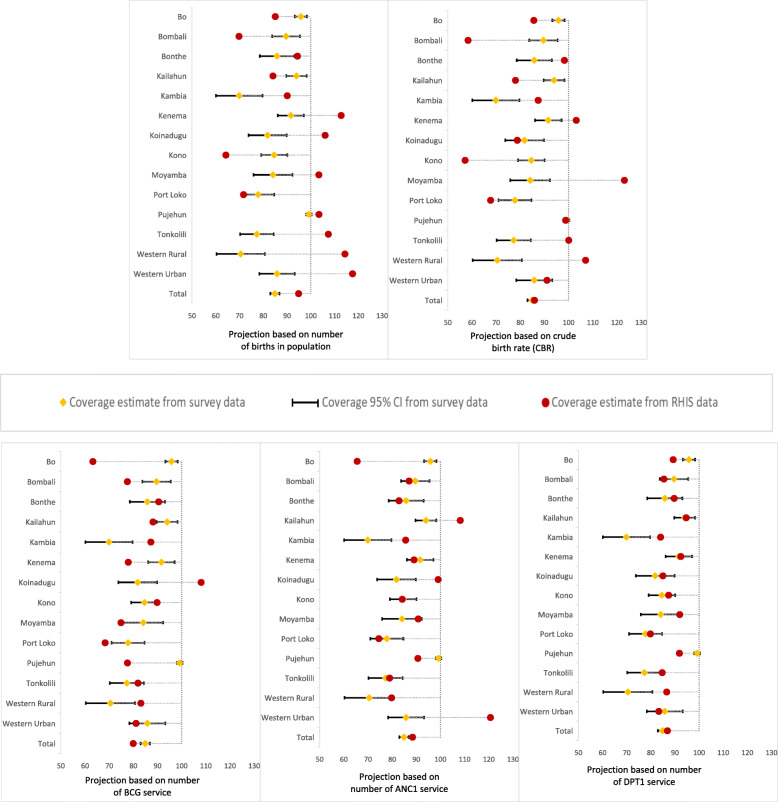
Level of agreement of DPT3 coverage estimates (%) between RHIS data and 2017 MICS, by district, using five different methods for calculating denominators

Using the census- and health service-based denominators, we computed four coverage estimates for ANC1 (Fig. 1 and Table A5), five coverage estimates for institutional delivery (Fig. 2 and Table A6) and DPT3 (Fig. 3 and Table A7). Additionally, we calculated the coverage rate with 95% confidence interval for each indicator at national and district levels using 2017 MICS data. We based the survey estimates on live births in the 2 years preceding the survey. The RHIS data covered the period 2014 to 2018, permitting calculation of the coverage estimates for the same reference period. Sierra Leone is one of the few countries for which the survey sample allowed computation of valid coverage estimates at district level. We assessed the level of agreement between the RHIS and the survey estimates at national and district levels. Coverage estimates from RHIS were considered consistent when their values were in-between the 95% confidence interval from the survey data. Moreover, we assessed the consistency over time between the survey national coverage and coverage estimates from census- and service-based methods (Figure A4 in annex).

Analyses were performed in MS Excel 2013 and Stata 14 SE [26] using data compiled from countries’ DHIS2 databases.

## Results

### Level of agreement of ANC1 coverage

Figure 1 and Table A5 compare ANC1 coverage based on four different denominators to the coverage obtained using MICS data. At national level, denominator computation approaches based on CBR and DPT1 provided coverage measure close to the survey estimate. However, regardless of the denominator method used, the agreement between the RHIS coverage estimate of ANC1 and the survey estimate was generally poor at district level. For more than half of the districts, the census-based denominators yielded coverage estimates greater than 100%. This suggests data quality issues from either the numerator (over-reporting), the denominator (under-estimation) or both. The national coverage was 104.5% for birth-based method and only one district (Western Urban Area) had adequate agreement. CBR-based method did not show a good agreement for any district, and the overall coverage estimate (94.6%) was statistically different from the survey estimate (97.1, 95% CI: 96.4–97.7%).

Considering the health service methods, we found a significant gap between the coverage of ANC1 and the survey estimates for the method based on either DPT1 or BCG, although the size of the gap was smaller compared to census-based adjustment methods. In Kambia (93.7%) and Pujehun (99.5%) districts, there was good agreement of the method based on DPT1 with the survey estimates (94.6, 95% CI: 91.2–96.8% versus 98.8, 95% CI: 95.8–99.7%). Overall, data from Kambia district generated more consistent ANC1 coverage estimates for all projection methods.

Finally, the size and the direction of the gaps between the RHIS-based coverage and the survey estimate was neither similar nor consistent across districts. This suggests irregularity in errors across the districts, preventing the use of a constant correction factor across the board.

### Level of agreement of institutional delivery coverage

The institutional delivery coverage estimates showed similar patterns as ANC1, characterized by inconsistencies between RHIS and the survey-based coverage estimates for most districts, and regardless of the denominator calculation method (Fig. 2 and Table A6). Only coverage estimate based on BCG showed consistency with the survey estimates. Coverage levels over 100% were more noticeable for census-based method. The discrepancies were all marked by overestimated RHIS-based coverage from Western Area Urban district, irrespective of the projection methods. Coverage estimates were 94.5 and 85.5% for birth- and CBR-based denominators compared to the survey 76.2% (95% CI: 74.0–78.4%) nationally. BCG-based denominator coverage was 79.6% while national estimates were 88.1 and 86.5% using ANC1- and DPT1-based denominators.

The institutional delivery coverage from BCG-based method also provided better levels of agreement at district level among all methods. We found an agreement for five districts out of fourteen (Kailahun, Kenema, Kono, Pujehun, and Western Rural) while three same districts fell within the 95% confidence interval of the survey coverage rates for both ANC1 and DPT1 service-based methods (Koinadugu, Kono, and Western Area Rural). Lower levels of agreement were observed for census-based methods as two districts (Bombali, and Bonthe) and one district (Bonthe) had coverage similar or close to estimates generated from the survey data. The districts of Kono, Koinadugu and Western rural for which we observed good agreement were also characterized by similar coverage rates across all health service-based methods (ANC1, BCG, and DPT1). The district of Bonthe only had consistent estimates across both census-based methods.

### Level of agreement of DPT3 coverage

Compared to ANC1 and institutional delivery estimates, there was better agreement of DPT3 estimates, primarily for health service-based methods. We found consistent estimate with the survey estimates at national level for CBR-based method, and a very small gap but statistically significant for ANC1, and DPT1-based methods (Fig. 3 and Table A7). DPT1 service-based method was the most appropriate method to calculate DPT3 coverages estimates as we found good agreement for more than half of the districts (8/14) and the national level coverage (86.9%) is close to the coverage from the survey (84.9, 95% CI: 82.8–86.7%). ANC1-based method generated consistent coverage estimates for less than half of the districts (6/14), and the national coverage (88.5%) was statistically different but close to the survey estimate (84.9, 95% CI: 82.8–86.7%). On the other hand, BCG-based method did not provide estimates with good agreement as observed for DPT1 and ANC1; only two districts had coverage estimates falling within the survey confidence interval. We observed consistent coverage estimates for a few districts across health service-based methods (Bombali, Bonthe, Kenema, and Tonkolili).

We found a poor agreement for coverage estimates calculated from both census-based methods. Using projected live births as denominator there was only one district (Port Loko) with consistent estimate. Interestingly, denominators based on crude birth rate (CBR) method showed good agreement at national level but poor agreement at district level. The national coverage from RHIS was 85.9%, falling within the 95% confidence interval of the survey estimate (84.9, 95% CI: 82.8–86.7%). However, only two districts met the agreement criteria.

## Discussion

The objective of the study was to generate coverage estimates for maternal and child health indicators from routine health facility data and assess the accuracy. We computed coverage of ANC1, institutional delivery, and DPT3 immunization using five different methods of estimating the denominator for Sierra Leone. These include two census-based methods and three health service-based methods. We compared the resulting estimates to those from the recent 2017 MICS at national and district levels for the same reference period. We also assessed the numerators for data quality and adjusted them for incompleteness of reporting. We found that while some approaches produce good agreement at national level, there was generally poor agreement at district level.

Census data have the advantage to provide population-based data which are usual sources to calculate the population in need of a health service. However, censuses are not conducted on a regular basis – every 10 years recommended – requiring the use of projections. Projections often lead to inaccuracies since the longer the census, the less accurate the projections. The challenges are related primarily to the projection assumptions about population growth, the changes over time in fertility, mortality, or migration. Furthermore, there is more uncertainty and inaccuracy for smaller geographies like districts as projection assumptions more often are made using national and constant estimates over time. CBR-based method generated a DTP3 national coverage (85.9%) consistent with the survey (84.9, 95% CI: 82.8–86.7%), but only two districts met the agreement criteria. This can be explained by the fact that districts with overestimated coverage rates offset the underestimated rates from others, leading to an average coverage rate close to the survey estimate at national level. If fertility and mortality are key underlying factors of population growth and change nationwide, recent or seasonal population movements may have large impact on population size and structure locally. Fertility and population growth assumptions at subnational level are usually made based on national estimates and constant values over time and across subnational units.

Variations in the place of care-seeking may lead to inaccurate coverage estimates when routine facility data are used. Indeed, it is not unusual that people seek care/service from a health district outside their district of residence. This would create an overcount or undercount in the numerators as well as inconsistency between individuals in need of the service (denominator) and those who received it in that district. This mismatch is one of the common issues that explains coverage rates over 100% as we observed for the two census-based methods. Overestimated coverage rates may also be due to age eligibility criteria. This is common with vaccination service for which children who received a vaccine at an older age are included in the coverage numerator while the denominator targets a narrower age group. ANC1 had the higher number of district coverage over 100%. This may suggest an over-reporting of the number of ANC1 services as a result of misclassification of antenatal care contacts or counting some higher order of ANC visits as first visit. A few studies highlighted the impact of Performance-based financing (PBF) or pay-for-performance (P4P) programs, using incentives for health workers, on health performance and data quality. Although output-based payment programs helped improve service provision, quality of care and the overall health system performance in certain settings, they may still have perverse consequences like incentives for overreporting, false reporting, or discrepancy between reported and actual coverage [27–31].

The inaccuracy of the denominator is one of the common reasons for coverage levels over 100%. Denominator inaccuracies may result from inaccurate estimate of population growth and fertility assumptions in population projections at national and notably subnational levels [2, 32]. These are potential explanations of the poor level of agreement of the coverage estimates calculated from the census-based methods and highlighted the persistent challenge of using census-based methods to derive coverage denominators. Health service-based methods appeared to perform better than census-based methods. This can be explained by the fact that the errors are more likely cancel out between the numerator and denominator. This is not the case for census-based method where the numerator and the denominator come from different sources, and data quality checks and correction focus on the numerator (RHIS data). However, there are a few challenges concerning the quality of routine health facility data and calculation methods.

None of the service-based methods clearly emerged as an alternative to deriving a denominator for ANC1 coverage estimates. However, denominators based on BCG service data generated consistent institutional delivery coverage rates in some districts. We found good DPT3 coverage concordance using denominators based on ANC1 service data, and better consistency using DPT1 service data. The quality of routine facility data has substantially improved over time as a result of improved organization and governance of the RHIS, increased funding, and the introduction of DHIS2 platform, making it an alternative data source to calculate health statistics besides facility surveys and household surveys [2, 8, 9, 33]. However, there are still data quality challenges along with the urgent need of refining methods for health service-based denominator calculation.

Coverage and denominator calculation based on health service data still require the use of assumptions derived from household survey. As examples, the calculation of the number of pregnancies from the adjusted number of ANC1 services requires an adjustment factor for non-use of ANC1 service obtained from household surveys. Similarly, stillbirth and neonatal rates from surveys are necessary to derive the expected number of live births and infants. The accuracy of the assumptions is also dependent on the time gap between the survey estimates and the health-facility-based estimates. Household surveys have also sampling errors and there are often data quality issues which make their use questionable for adjusting health service-based denominators. Furthermore, the lower administrative unit in household surveys is generally different to the lower unit for facility data – typically the district. Although we applied recommended data quality metrics, [23] we may not have identified and addressed all data quality issues.

The completeness of reporting constitutes one of the major data quality issues, although countries made recent improvements in that respect [34, 35]. This was particularly the case for subnational estimates. Sierra Leone had an overall good completeness of reporting rate above the WHO suggested threshold of 80%, [23] but we found subnational completeness rates below this threshold and a difference of more than 20 percentage points from the national average rate. This reinforces the need to go beyond national level and check data quality at subnational level as well. Adjustment for incomplete reporting helps tackle completeness issues, [2] but most countries do not account for this correction in their health statistics. Our coverage estimates were calculated accounting for correction of incomplete reporting, but further improvements are necessary for correction and adjustment of reported data, especially at subnational levels. The fact that the size and the direction of the gaps between the RHIS-based coverage and the survey estimate was not consistent across districts suggests irregularity in errors across the districts, preventing the use of a constant correction factor across the board. District capacities through human resources, training, supervision and access to adequate equipment and technologies are also critical factors in looking for possible explanations of variations across districts. Country and particularly district capacities are still limited for good data quality checks, adjustment and production of credible statistics [10]. This underlines the need to strengthen district capacities considering together human, technical, organizational factors for improving the routine health information system. The promotion of culture of data is also a crucial factor for fulfilling this objective [5, 10, 33].

Incomplete reporting was adjusted by type of health service, but the adjustments were based on an average national correction value by service. It would be worthwhile determining and using district-specific adjustment factor, since districts differ by several factors, including the category of facilities, type of facility management, level of urbanity, stock-outs issues, etc. Western Area Urban (83.8%) alongside Western Area Rural (86.6%) districts – representing the Western Area – had the lower reporting rates of institutional delivery services. Moreover, the lowest reporting rate of vaccination service was in Western Urban Area district (69.1%) and far away from the national average (89.3%). Urban districts have usually lower reporting rates since they comprise more private facilities and hospitals which often have poor reporting to the routine system. Of the 113 non-public health facilities in the RHIS, 35% were from Western Urban Area district, 14% in Western Area Rural district and the other half distributed among the remaining twelve districts. The Western Area Urban is the most populous and densely populated district in Sierra Leone covering about 15% of the population [21]. It includes the capital city Freetown along with most private facilities and hospitals. On the other hand, the adjustment procedure accounted for only health facilities included in the RHIS; in total, 1534 health facilities were included nationwide.

Finally, the adjustment and correction that we used focus on the completeness and less on the accuracy and consistency of reported data. We found that two services (BCG and DPT1) with a similar reporting rate and the same adjustment factor for incomplete reporting generated denominators and coverage estimates with different levels of agreement due primarily to the content of reported data for each service. This confirms the need to also assess and correct the accuracy of the reported data along with the completeness of reporting.

The study highlights the challenges in determining an appropriate denominator for coverage statistics from routine health facility data using Sierra Leone as a case study. Using findings from one country and coverage estimates for a given year may be a limitation for the generalizability of the findings to other countries. Yet, data quality checks showed poor quality in most countries, suggesting that some of the findings from this case study may apply to other countries. However, it will be worthwhile applying the same study design to other countries to draw meaningful conclusion with respect to generalizability of the findings.

Moreover, when the differences between a census-based and service-based estimates are small and credible, it is desirable to use the census-based estimates, particularly for national or subnational units higher than the health district [10]. We also highlighted the need for considering individual service analysis and individual district analysis to choose a denominator. In addition, a denominator may work for a specific service only, while consistency at national level does not necessarily mean the denominator works at the district or subnational level.

## Conclusion

A key challenge for measuring health service coverage from routine health facility data is to accurately estimate the denominator. Although we found better consistency of coverage estimates from health service-based methods compared to census-based methods, no single method clearly emerged across the board. Furthermore, a good population projection or national coverage estimate does not always translate into consistent outcomes at district level and highlights the challenge in determining accurate assumptions and population projections at subnational level. The choice of a denominator is determined by multiple factors and considerations: It depends on quality arguments based on a systematic data quality checks and correction. That implies correction of accuracy and consistency, along with rigorous adjustment for incomplete reporting considering district specificities (predominant type of facilities and management, level of urbanity, stock-outs, and other contextual factors) on a yearly basis. Data quality check and correction as well as the calculation of denominator must be carried out transparently and systematically. That also entails improving logistics, human resources, and capacity building namely for data quality checks, analysis, use, interpretation, and dissemination at both district and central levels. A promotion of data culture combined to an improved commitment and leadership of health district and regional teams are also likely to contribute to generate accurate and valuable statistics for planning and evaluation of health interventions locally and nationwide.

## Supplementary Information

**Additional file 1.**

## Data Availability

The dataset cannot be made publicly available, but analysis materials and selected data are available from the corresponding author on reasonable request. This article has been published as part of BMC Health Services Research Volume 21 Supplement 1 2021: Health facility data to monitor national and subnational progress. The full contents of the supplement are available at https://bmchealthservres.biomedcentral.com/articles/supplements/volume-21-supplement-1.

## Abbreviations

ANC: Antenatal care
BCG: Bacille de Calmette and Guerin
CBR: Crude Birth Rate
DHIS2: District Health Information Software 2
DHS: Demographic and Health Survey
DPT: Diphtheria-Pertussis-Tetanus
MICS: Multiple Indicators Cluster Survey
RHIS: Routine Health Information Systems
SD: Standard deviation
WHO: World Health Organization
