# Supplementary material for: Measuring coverage of maternal and child health services using routine health facility data: a Sierra Leone case study
Source: BMC Health Serv Res. 2021 Sep 13;21(Suppl 1):547. doi: 10.1186/s12913-021-06529-7 (PMC8435364; doi:10.1186/s12913-021-06529-7)
Supplement: Supplementary file 1 — Additional file 1. [file 12913_2021_6529_MOESM1_ESM.docx]

**Additional files**

**Annex tables and figures**

**Table A1.** General characteristics of main sources of data in West and Central Africa countries

| **Country** | **Latest census**  **Year** | **Latest household survey** | | | | **Latest RHIS data** | |
| --- | --- | --- | --- | --- | --- | --- | --- |
|  |  | **Survey-Year** | **Number of households** | **Number of sub-national units** | **Year** | | **Number of administrative units** |
| Benin | 2013 | DHS-2017/18 | 14156 | 12 Departments | 2018 | | 22 Districts |
| Burkina Faso | 2006 | DHS-2010 | 14424 | 13 Regions | 2018 | | 34 Districts |
| Cabo Verde | 2010 | DHS-2005 | 5712 | 12 Islands/ Municipalities | 2018 | | 70 Districts |
| Cameroon | 2005 | DHS-2018 | 11710 | 12 Regions | 2018 | | 180 Districts |
| CAR | 2003 | MICS-2010 | 11756 | 17 Prefectures | 2018 | | 16 Districts |
| Chad | 2009 | DHS-2014/15 | 17233 | 21 Regions | - | | - |
| Congo | 2007 | MICS-2014/15 | 12811 | 12 Departments | 2018 | | 12 Regions |
| Cote d'Ivoire | 2014 | MICS-2016 | 11879 | 11 Regions | 2018 | | 81 Districts |
| DRC | 1984 | MICS-2017/18 | 20792 | 26 Provinces | 2018 | | 519 Districts |
| Gabon | 2013 | DHS-2012 | 9755 | 10 Provinces | 2018 | | 51 Districts |
| Ghana | 2010 | MICS-2017/18 | 12886 | 10 Regions | 2018 | | 260 Districts |
| Guinea | 2014 | DHS-2018 | 7912 | 8 Regions | 2018 | | 38 Regions |
| Guinea-Bissau | 2009 | MICS-2014 | 6601 | 9 Regions | 2018 | | 111 Districts |
| Liberia | 2008 | DHS-2013/14 | 9333 | 15 Counties | 2018 | | 92 Districts |
| Mali | 2009 | DHS-2018 | 9510 | 9 Regions | 2018 | | 74 Districts |
| Niger * | 2012 | DHS-2012 | 10750 | 8 Regions | 2018 | | 72 Districts |
| Nigeria | 2006 | DHS-2018 | 40427 | 37 States | 2018 | | 145 Districts |
| Senegal † | 2013 | DHS-2016/17/18 | 17409 | 14 Regions | 2018 | | 77 Districts |
| Sierra Leone | 2015 | MICS-2017 | 15309 | 14 Districts | 2018 | | 14 Districts |
| Togo | 2010 | MICS-2017 | 7916 | 7 Regions | 2018 | | 41 Districts |
|  |  |  |  |  |  | |  |
| **Median** | **2010** | **2017** | **11818** | **12** | **2018** | | **72** |

** DHS 2017, but report not released and data not distributed*

*† Continuous DHS*

**Table A2.** List of coverage indicators available from the RHIS data analyzed

| **Category** | **Indicator** | **Definition** |
| --- | --- | --- |
| Maternal health | Antenatal care from a health facility (ANC1) | Proportion of pregnant women who had at least one contact with a health facility during a calendar year for reasons related to antenatal care |
| Maternal health | Institutional delivery | Proportion of women who had a live birth in a health facility during a calendar year |
| Child health | BCG immunization | Proportion of infants born during a calendar year who received one dose of *Bacille de Calmette and Guerin* (BCG) vaccine in a health facility |
| Child health | Penta/DPT3 immunization | Proportion of infants born during a calendar year who received three doses of Pentavalent/diphtheria-pertussis-tetanus vaccine in a health facility |
| Child health | Measles immunization | Proportion of infants born during a calendar year who received one dose of measles vaccine in a health facility |

|  |  |
| --- | --- |
|  |  |

*Red dots correspond to median scores of completeness of reporting*

**Figure A1.** Median score of completeness of reporting (%) of ANC, vaccination, institutional delivery and OPD

**Table A3.** Percentage of districts with good consistency of reported data in 2018 compared to the 3 preceding years

| **Country** | **CPN1** | **DTP1** | **OPD** | **Average score** |
| --- | --- | --- | --- | --- |
|  | **% districts with good consistency (Z-score<2)** | **% districts with good consistency (Z-score<2)** | **% districts with good consistency (Z-score<2)** | **% districts with good consistency (Z-score<2)** |
| Benin | 41 | 53 | 41 | 45 |
| Burkina | 56 | 70 | 78 | 68 |
| Cabo Verde | 30 | 50 |  | 40 |
| CAR | - | 47 | - | 47 |
| Cameroun | 52 | 4 | 59 | 38 |
| Congo | 100 | 25 |  | 63 |
| Cote d'Ivoire | 51 | - | 73 | 62 |
| DRC | - | - | - | - |
| Gabon | 73 | 50 | 41 | 55 |
| Ghana | 52 | 52 | 48 | 51 |
| Guinea | 5 | 8 | 45 | 19 |
| Guinea-Bissau | 78 | - | 55 | 66 |
| Liberia | 52 | 71 | 69 | 64 |
| Mali | - | - | 57 | 57 |
| Niger | 32 | 49 | 25 | 35 |
| Nigeria | 51 | 62 | 56 | 56 |
| Senegal | 71 | 51 | 66 | 63 |
| Sierra Leone | 29 | 36 | 71 | 45 |
| Togo | 49 | 73 | 76 | 66 |
|  |  |  |  |  |
| **Median** | **51** | **50** | **57** | **55** |

*Color scale indicates good data quality for dark green color while light green color corresponds to poor data quality.*

**Table A4.** Numerator internal consistency: Difference between ratio of expected and ratio of reported for ANC1 & DPT1 and DPT3 (%)

| **Country** | **Difference ratio 2018** | | **Percentage districts difference ratio ANC1 - DPT1** | | | **Percentage districts difference ratio DPT1 - DPT3** | | | **Average score difference ratio ANC1 - DPT1 & difference ratio DPT1 - DPT3** |
| --- | --- | --- | --- | --- | --- | --- | --- | --- | --- |
|  | **ANC1 - DPT1** | **DPT1 - DPT3** | **more or equal to 15%** | **in-between 5-15%** | **less or equal to 5%** | **more or equal to 15%** | **in-between 5-15%** | **less or equal to 5%** | **less than 15%** |
| Benin | 23 | 7 | 71 | 18 | 12 | 35 | 21 | 44 | **47** |
| Burkina Faso | 5 | 2 | 37 | 41 | 21 | 0 | 23 | 77 | **81** |
| Cabo Verde |  |  | 59 | 23 | 18 | 9 | 32 | 59 | **66** |
| CAR | 5 | 113 | 92 | 0 | 8 | 100 | 0 | 0 | **4** |
| Cameroon | 23 | 0 | 72 | 17 | 11 | 18 | 46 | 37 | **55** |
| Congo | 79 |  | 100 | 0 | 0 | 91 | 9 | 0 | **5** |
| Cote d'Ivoire | 2 | 28 | 52 | 31 | 17 | 77 | 17 | 6 | **36** |
| DRC | - | - | - | - | - | - | - | - | **-** |
| Gabon | 9 | 6 | 77 | 14 | 9 | 37 | 37 | 25 | **43** |
| Ghana | 25 | 9 | 62 | 24 | 14 | 24 | 52 | 24 | **57** |
| Guinea | 46 | 62 | 89 | 3 | 8 | 100 | 0 | 0 | **5** |
| Guinea-Bissau | 7 | 1 | - | - | - | - | - | - | **-** |
| Liberia | 15 | 22 | 69 | 22 | 9 | 77 | 12 | 11 | **27** |
| Mali | 40 | 15 | 85 | 8 | 7 | 65 | 26 | 9 | **25** |
| Niger | 24 | 19 | 78 | 18 | 4 | 58 | 22 | 19 | **32** |
| Nigeria | 198 | 76 | 100 | 0 | 0 | 100 | 0 | 0 | **0** |
| Senegal | 39 | 2 | 88 | 5 | 7 | 4 | 39 | 57 | **54** |
| Sierra Leone | 4 | 5 | 21 | 57 | 21 | 7 | 43 | 50 | **86** |
| Togo | 8 | 1 | 41 | 39 | 20 | 0 | 22 | 78 | **79** |
|  |  |  |  |  |  |  |  |  |  |
| **Median** | **23** | **8** | **72** | **18** | **9** | **37** | **22** | **24** | **43** |

*Color scale indicates good data quality for green color while red color corresponds to poor data quality.*

**Table A5.** ANC1 Coverage estimates from household survey and absolute difference of ANC1 coverage estimates using RHIS data with the household survey coverage estimates

|  | **Coverage estimates from household survey, 2017 MICS (%)** | | | **Absolute difference of RHIS coverage estimates from survey estimates, using the different methods for calculating denominators (%)** | | | |
| --- | --- | --- | --- | --- | --- | --- | --- |
| **Country** | **Estimate** | **SE** | **95% CI** | **Birth-method** | **CBR- method** | **BCG-method** | **DPT1-method** |
| Bo | 99.1 | 0.5 | [97.2-99.7] | 28.3 | 29.2 | -4.3 | 34.6 |
| Bombali | 98.7 | 0.9 | [94.8-99.7] | -22.2 | -34.7 | -13.7 | -5.1 |
| Bonthe | 96.3 | 1.3 | [92.8-98.2] | 15.6 | 20.2 | 10.9 | 10.0 |
| Kailahun | 99.2 | 0.5 | [97.5-99.7] | -22.5 | -28.0 | -18.8 | -12.9 |
| Kambia | 94.6 | 1.4 | [91.2-96.8] | 5.8 | 2.8 | 2.6 | -0.9 |
| Kenema | 98.5 | 0.8 | [95.6-99.5] | 26.5 | 15.9 | -12.2 | 3.9 |
| Koinadugu | 92.9 | 2.6 | [86.0-96.6] | 9.2 | -17.0 | 11.2 | -11.0 |
| Kono | 99.2 | 0.5 | [97.4-99.8] | -23.9 | -32.2 | 6.1 | 3.2 |
| Moyamba | 95.5 | 1.2 | [92.6-97.4] | 16.3 | 37.6 | -14.5 | 4.0 |
| Port Loko | 94.2 | 1.8 | [89.5-96.9] | -2.4 | -7.5 | -6.7 | 8.0 |
| Pujehun | 98.8 | 0.8 | [95.8-99.7] | 13.2 | 8.2 | -14.8 | 0.7 |
| Tonkolili | 95.3 | 1.5 | [91.3-97.5] | 34.4 | 25.6 | 3.7 | 7.1 |
| Western Rural | 98.5 | 0.7 | [96.1-99.4] | 43.0 | 33.9 | 4.3 | 8.6 |
| Western Urban | 97.1 | 1.1 | [94.1-98.6] | -1.0 | -22.6 | -30.8 | -29.0 |
| Total | 97.1 | 0.3 | [96.4-97.7] | 7.4 | -2.5 | -9.1 | -1.4 |

*SE: Standard errors; 95% CI: 95% confidence interval*

*Color scale indicates good agreement (lower difference) for green color while orange color corresponds to poor agreement (higher difference). Dark green corresponds to difference between RHIS estimates and survey estimates less than 5%, light green 5-9%, yellow 10-19%, and orange 20% and more.*

**Table A6.** Institutional delivery coverage estimates from household survey and absolute difference of ANC1 coverage estimates using RHIS data with the household survey coverage estimates

|  | **Coverage estimates from household survey, 2017 MICS (%)** | | | **Absolute difference of RHIS coverage estimates from survey estimates, using the different methods for calculating denominators (%)** | | | | |
| --- | --- | --- | --- | --- | --- | --- | --- | --- |
| **Country** | **Estimate** | **SE** | **95% CI** | **Birth-method** | **CBR- method** | **BCG-method** | **ANC1-method** | **DPT1-method** |
| Bo | 96.4 | 1.1 | [93.5-98.0] | 20.9 | 21.7 | -9.2 | -6.0 | 26.7 |
| Bombali | 76.7 | 3.5 | [69.2-82.9] | 10.3 | -3.9 | 19.9 | 31.8 | 29.8 |
| Bonthe | 91.8 | 2.6 | [84.9-95.7] | -4.9 | -1.3 | -8.5 | -15.5 | -9.2 |
| Kailahun | 93.9 | 2.4 | [87.3-97.1] | -7.1 | -13.4 | -3.0 | 17.8 | 3.8 |
| Kambia | 53.9 | 5.1 | [43.8-63.7] | 42.5 | 39.6 | 39.4 | 37.7 | 36.0 |
| Kenema | 91.0 | 2.8 | [83.7-95.2] | 32.2 | 21.8 | -5.9 | 6.3 | 10.0 |
| Koinadugu | 76.4 | 5.0 | [65.3-84.7] | 10.0 | -12.2 | 11.7 | 4.3 | -7.1 |
| Kono | 74.6 | 5.1 | [63.4-83.2] | -15.1 | -21.7 | 8.6 | 3.4 | 6.3 |
| Moyamba | 60.3 | 7.0 | [46.2-72.9] | 66.1 | 90.2 | 31.3 | 50.8 | 52.2 |
| Port Loko | 56.5 | 3.8 | [48.9-63.8] | 34.0 | 29.0 | 29.7 | 37.5 | 44.3 |
| Pujehun | 92.3 | 3.1 | [83.4-96.6] | 33.9 | 28.2 | 2.3 | 18.3 | 19.8 |
| Tonkolili | 61.4 | 4.9 | [51.5-70.4] | 51.4 | 43.7 | 24.7 | 21.6 | 27.6 |
| Western Rural | 67.0 | 5.6 | [55.3-77.0] | 26.7 | 20.7 | 1.1 | -1.6 | 3.9 |
| Western Urban | 79.8 | 2.7 | [73.9-84.6] | -17.6 | -31.6 | -36.9 | -15.9 | -35.7 |
| Total | 76.2 | 1.1 | [74-78.4] | 18.3 | 9.3 | 3.4 | 11.9 | 10.3 |

*SE: Standard errors; 95% CI: 95% confidence interval*

*Color scale indicates good agreement (lower difference) for green color while orange color corresponds to poor agreement (higher difference). Dark green corresponds to difference between RHIS estimates and survey estimates less than 5%, light green 5-9%, yellow 10-19%, and orange 20% and more.*

**Table A7.** DPT3 Coverage estimates from household survey and absolute difference of DPT3 coverage estimates using RHIS data with the household survey coverage estimates

|  | **Coverage estimates from household survey, 2017 MICS (%)** | | | **Absolute difference of RHIS coverage estimates from survey estimates, using the different methods for calculating denominators (%)** | | | | |
| --- | --- | --- | --- | --- | --- | --- | --- | --- |
| **Country** | **Estimate** | **SE** | **95% CI** | **Birth-method** | **CBR- method** | **BCG-method** | **ANC1-method** | **DPT1-method** |
| Bo | 95.8 | 1.3 | [92.4-97.7] | -10.8 | -10.2 | -32.5 | -30.2 | -6.5 |
| Bombali | 89.6 | 3.0 | [82.0-94.2] | -19.8 | -31.2 | -12.1 | -2.6 | -4.2 |
| Bonthe | 85.8 | 3.7 | [77.1-91.6] | 8.6 | 12.5 | 4.7 | -2.9 | 3.9 |
| Kailahun | 94.0 | 2.2 | [88.1-97.1] | -9.9 | -16.0 | -5.9 | 14.2 | 0.7 |
| Kambia | 69.9 | 5.0 | [59.2-78.8] | 20.2 | 17.5 | 17.3 | 15.7 | 14.2 |
| Kenema | 91.6 | 2.8 | [84.1-95.8] | 21.2 | 11.6 | -13.7 | -2.5 | 0.8 |
| Koinadugu | 81.8 | 4.1 | [72.4-88.6] | 24.2 | -3.0 | 26.3 | 17.3 | 3.2 |
| Kono | 84.6 | 2.8 | [78.2-89.3] | -20.3 | -27.4 | 5.2 | -0.4 | 2.8 |
| Moyamba | 84.1 | 4.2 | [74.1-90.7] | 19.3 | 39.0 | -9.2 | 6.7 | 7.9 |
| Port Loko | 77.8 | 3.5 | [70.2-83.9] | -6.1 | -10.1 | -9.5 | -3.3 | 2.1 |
| Pujehun | 99.2 | 0.6 | [96.7-99.8] | 4.2 | -0.4 | -21.6 | -8.5 | -7.3 |
| Tonkolili | 77.3 | 3.6 | [69.5-83.6] | 30.1 | 22.8 | 4.6 | 1.7 | 7.4 |
| Western Rural | 70.5 | 5.2 | [59.3-79.7] | 43.9 | 36.6 | 12.6 | 9.3 | 16.1 |
| Western Urban | 85.8 | 3.8 | [76.7-91.8] | 31.8 | 5.3 | -4.7 | 34.9 | -2.5 |
| Total | 84.9 | 1.0 | [82.8-86.7] | 10.0 | 1.0 | -5.0 | 3.6 | 2.0 |

*SE: Standard errors; 95% CI: 95% confidence interval*

*Color scale indicates good agreement (lower difference) for green color while orange color corresponds to poor agreement (higher difference). Dark green corresponds to difference between RHIS estimates and survey estimates less than 5%, light green 5-9%, yellow 10-19%, and orange 20% and more.*


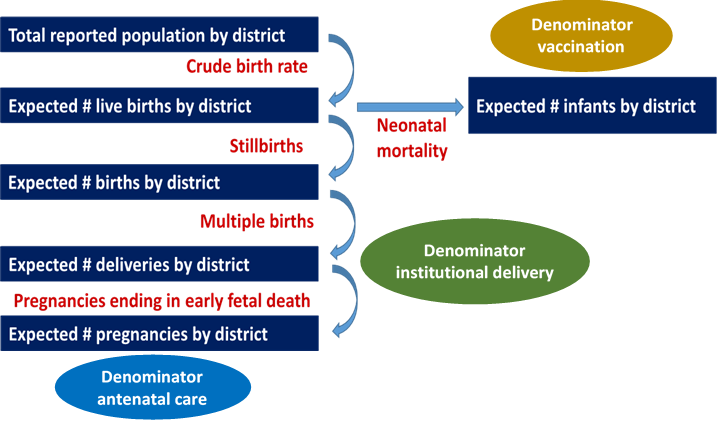


**Figure A2.** Summary of the steps of denominators calculation from census-based methods


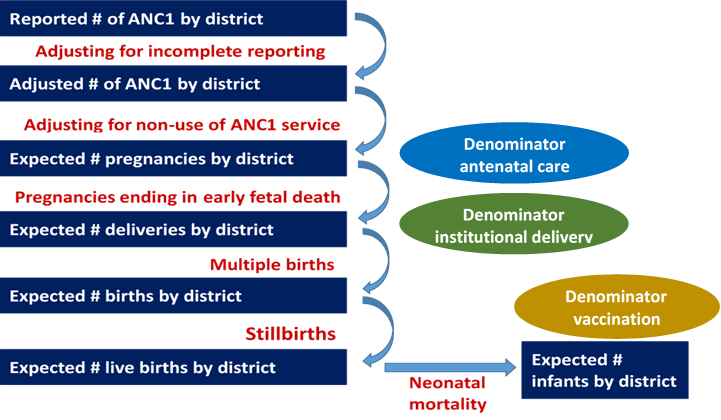


**Figure A3.** Summary of the steps of denominators calculation from health service-based method using the example of ANC1 data

| 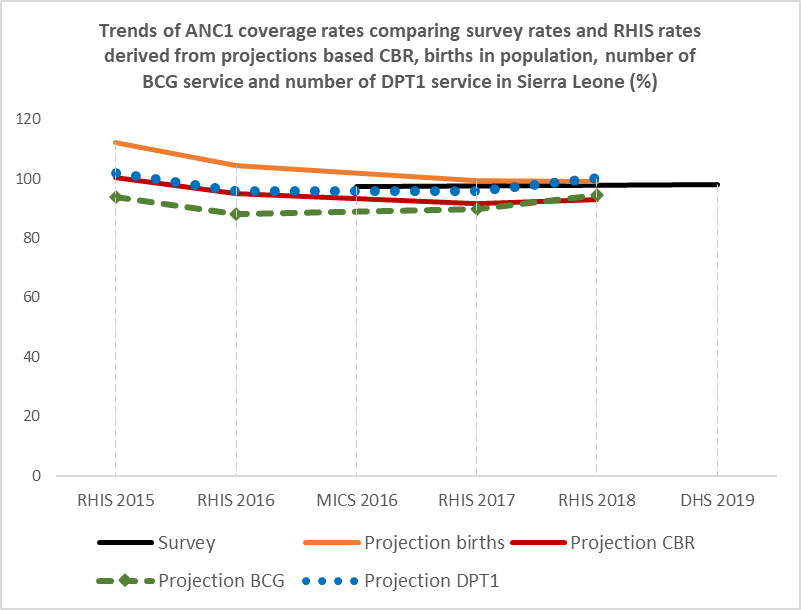 |
| --- |
|  |
| 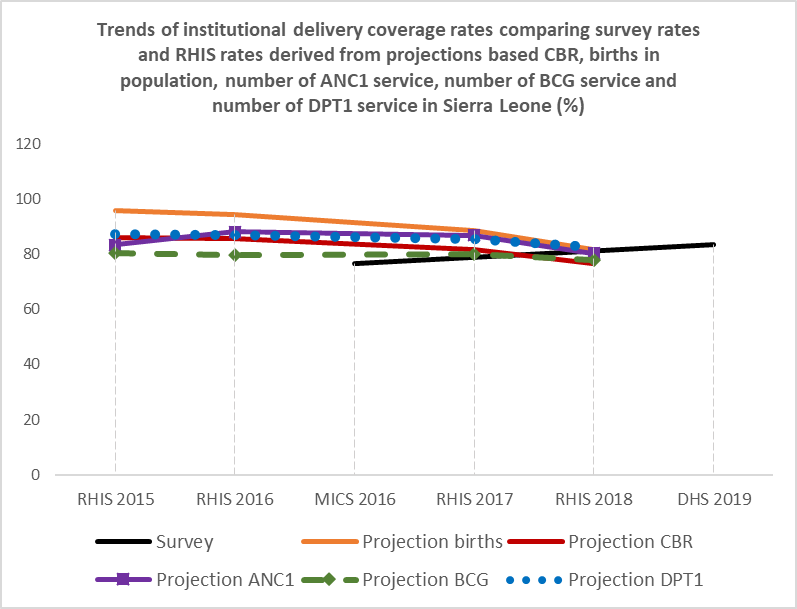 |
|  |
| 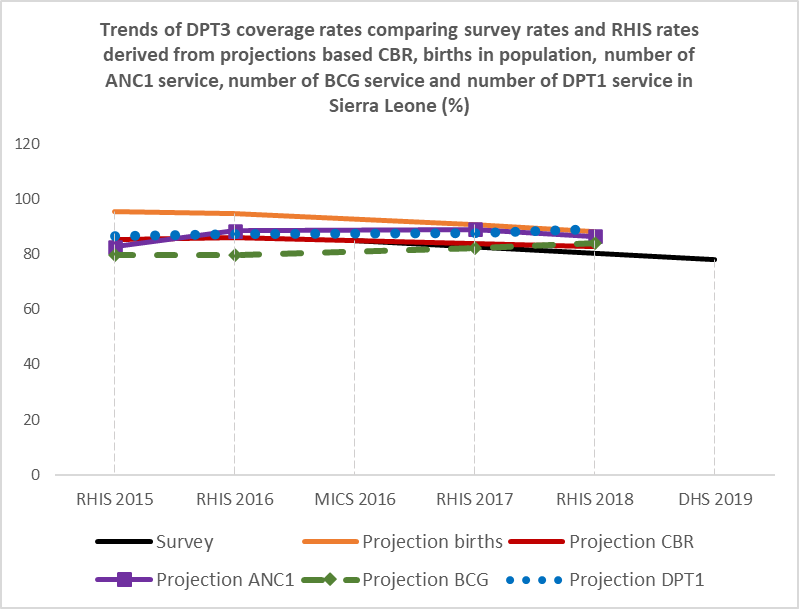 |

**Figure A4.** Trends of ANC1, institutional delivery and DPT3 coverage rates comparing survey rates and RHIS rates derived from projections based CBR, births in population, number of ANC1 service, number of BCG service and number of DPT1 service in Sierra Leone (%)
